# Kinetics of Drug Release from Clay Using Enhanced Sampling Methods

**DOI:** 10.3390/pharmaceutics14122586

**Published:** 2022-11-24

**Authors:** Ana Borrego-Sánchez, Jayashrita Debnath, Michele Parrinello

**Affiliations:** Center for Human Technologies, Italian Institute of Technology (IIT), Via Enrico Melen 83, 16152 Genoa, Italy

**Keywords:** drug release, praziquantel, clay minerals, computational calculations, enhanced sampling molecular dynamics, kinetics

## Abstract

A key step in the development of a new drug, is the design of drug–excipient complexes that lead to optimal drug release kinetics. Computational chemistry and specifically enhanced sampling molecular dynamics methods can play a key role in this context, by minimizing the need for expensive experiments, and reducing cost and time. Here we show that recent advances in enhanced sampling methodologies can be brought to fruition in this area. We demonstrate the potential of these methodologies by simulating the drug release kinetics of the complex praziquantel–montmorillonite in water. Praziquantel finds promising applications in the treatment of schistosomiasis, but its biopharmaceutical profile needs to be improved, and a cheap material such as the montmorillonite clay would be a very convenient excipient. We simulate the drug release both from surface and interlayer space, and find that the diffusion of the praziquantel inside the interlayer space is the process that limits the rate of drug release.

## 1. Introduction

One of the challenges in pharmacological studies is the design of new drug delivery systems that have a release profile able to reduce doses and minimize side effects. This implies developing new drug–excipient combinations. An example of current relevance is praziquantel. This is the drug of choice in the treatment of schistosomiasis, which is a widely spread but neglected tropical disease. Worldwide more than 700 million people are exposed to the parasite that carries this disease and 240 million are infected, mainly in the tropical and subtropical regions of developing countries [1,2,3,4,5]. Praziquantel is administered orally, but due to its low solubility high doses are needed to obtain effective concentrations in the blood [6,7,8,9,10]. This causes side effects and leads to drug resistance [11,12]. To overcome these limitations, scientific research is needed to improve the praziquantel aqueous solubility, for example, by using cheap excipients to keep the cost of the medicine low, which is a major issue in developing countries. In this sense, excipients based on clay minerals are attractive candidates. In fact, montmorillonite has been already used in pharmaceutical practice since it is abundant and has many advantageous properties. It is cheap, safe, non-toxic, biocompatible, and highly adsorbent, and it can encapsulate the drug in the nanosized interlayer spaces [13,14,15,16,17,18]. One of us has already experimentally studied the potential effect of using montmorillonite as an excipient and found that it increases praziquantel solubility [19,20].

Currently, knowledge on the interaction of organic molecules, such as praziquantel, with excipients that have complex structures such as pores, nanotubes or interlayers, is still being built up. The investigation of the mechanism of drug release would profit from the use of computational chemistry techniques that are able to investigate the drug–clay complexes. One of us has already performed static calculations using classical or quantum mechanical approaches [21]. In addition, investigations of the local dynamical interactions have been reported [21]. However, these latter studies were limited by the relatively small timescale explored. Since the drug release takes a time that exceeds what is possible, it is necessary to go beyond standard molecular dynamics (MD) simulations.

To calculate the rates, we apply and compare the performance of two enhanced sampling methods that are designed to overcome the timescale barrier. One is the Gaussian Mixture-Based Enhanced Sampling (GAMBES) [22]. The other is a variant of the On-the-fly Probability Enhanced Sampling (OPES) method, namely OPES flooding (OPES_f_) [23]. The OPES_f_ method has been carefully benchmarked in a ligand–protein system, for which there are accurate experimental data directly comparable with the computations [24]. In that study, a calculated residence time of 6.87 × 10^2^ s^−1^ was computed, which agrees with the experimental value of 6.00 ± 3.00 × 10^2^ s^−1^. This methodology, with such a level of accuracy, is of high relevance in the pharmaceutical field and it is therefore our objective to use it in the present work. We shall interpret our previous experiments on the praziquantel release from montmorillonite [19,20], quantifying the kinetics of the process.

Through the application of these enhanced sampling simulations to the release of praziquantel from the surface and the interlayer space of montmorillonite, we aim to present a viable computational strategy that could be applied in other drug release simulations.

## 2. Methods

We studied two different systems, in one, the drug is absorbed on the top surface of a montmorillonite, and in the other, the drug sits in between the layers. In the first case, the dynamics are fast and the release can be simulated with standard molecular dynamics. In the second case, the release time is too long to be simulated in this way, thus it requires the use of enhanced sampling methods. Here, we summarize the methods used in this second case.

Over the years, many enhanced sampling methods have been developed in the last few decades for the study of rare event processes (see, for instance, references: [25,26,27,28,29,30,31,32,33]). However, most of these methods were designed for calculating static properties, such as free energy differences. These methods and others, such as parallel tempering, alter the dynamics.

Luckily, some of these methods can be engineered to be able to compute the rates. This was made possible by the observation made by Grubmüller [34] and Voter [35], that dynamic properties can still be extracted from biased simulations that are accelerated by the addition of an external bias: $V\left(R\right)$ function of the atomic coordinates R provided that such bias is null the transition state region. In such a case, a simple relation links the physical time τ, the apparent escape time $\tau_{MD}$ and the bias deposited $V\left(R\right)$:

(1) $$\tau =\langle e^{\beta V\left(R\right)}\rangle_{V}\;\tau_{MD}$$

where the average is over the bias simulation and $\beta ={\left(k_{B}T\right)}^{-1}$ is the inverse Boltzmann factor.

Here we shall use enhanced sampling methods, in which the bias dependence on R is mediated via a set of functions of R. Depending on the method, these functions are referred to as descriptors $d\left(R\right)$ or as collective variables $s\left(R\right)$. For this type of bias, many suggestions have been made to design a potential that satisfies the Grubmüller and Voter conditions [33,36]. In this work, we use and compare two such enhanced sampling methods, Gaussian Mixture Based Enhanced Sampling (GAMBES) [22] and On-the-fly Probability Enhanced Sampling flooding approach (OPES_f_) [23], which ensure in a relatively simple way that no bias is added to the transition state. We outline here these two methods that differ in the way the bias is constructed. The description of the two methods is considerably simplified if we limit ourselves to describing how to use them to compute the escape times from the bound state, instead of reconstructing the full free energy landscape. We refer to the original literature for a full description of the two methods.

*GAMBES*

In GAMBES, one introduces $N_{d}$ descriptors $d\left(R\right)\;\equiv \;\left\{d_{p}\left(R\right),\;p=1,2\ldots N_{d}\right\}$ able to characterize the initial state, and performs an unbiased simulation in the bound state. From these data, the probability density $p\left(d\left(R\right)\right)$ is estimated by fitting the data to a Gaussian mixture [37]. A bias is then constructed using the relation $V\left(d\right)=\left(\frac{1}{\beta }\right)\log\left(P\left(d\right)+\epsilon \right)$ where ϵ is a smoothing parameter that also limits the amount of bias that is added. This is very helpful in making sure the conditions for the validity of Equation (1) are satisfied.

*OPES*

The OPES method is also based on building a bias, starting from an estimate of the probability distribution [23]. Rather than using descriptors, that can be very many, one uses (such as in umbrella sampling or metadynamics) a small number of collective variables s=s(R). However, in the spirit of metadynamics, the probability $P\left(s\right)$ is constructed on the fly as a linear combination of multivariate Gaussians. The bias is constructed in such a way as to modify, in a preassigned way ($P^{tg}\left(s\right)$), the s probability distribution. We shall refer to $P^{tg}\left(s\right)$ as the target distribution. Here, we shall use as target distribution the so called well-tempered one, $P\left(s\right)\propto {\left[P\left(s\right)\right]}^{\frac{1}{\gamma }}$ where the parameter γ>1 regulates the broadening of the target distribution.

The estimate of the probability is periodically updated and at iteration *n* is written as $P_{n}\left(s\right)=\frac{\sum_{k}^{n}w^{k}G\left(s,s_{k}\right)}{\sum_{k}^{n}w^{k}}$, where $G\left(s,s_{k}\right)$ are multivariate Gaussian kernels centered at the value assumed by the collective variable at the previous steps *k*, the weights $w_{k}=e^{\beta V_{k-1}\left(s_{k}\right)}$ are computed from the bias at step k−1, and bias is updated as $V_{n}\left(s\right)=\left(1-\frac{1}{\gamma }\right)\frac{1}{\beta }\log\;\left(\frac{P_{n}\left(s\right)}{Z_{n}}+\epsilon \right)$. In the bias expression ϵ is a smoothing parameter such as the one used in GAMBES and $Z_{n}$ is a normalizing factor.

To calculate the rates, we use the OPES flooding variant (OPES_f_) that is a modification of OPES designed to avoid depositing bias in the transition region [23,36]. As in GAMBES, the ϵ is used to avoid overflowing the basin. Furthermore, the parameter EXCLUDED_REGION can be used to prevent OPES_f_ from depositing bias in the preassigned region of the configurational space, corresponding to the transition state.

In both cases, several calculations are started in the bound basin and the statistical distribution of the exit times τ are analyzed using the Kolmogorov–Smirnov test, to ensure that is Poissonian as appropriate for a rare event scenario. 

### 2.1. The Model

#### 2.1.1. The Surface Model System

**Figure 1:**
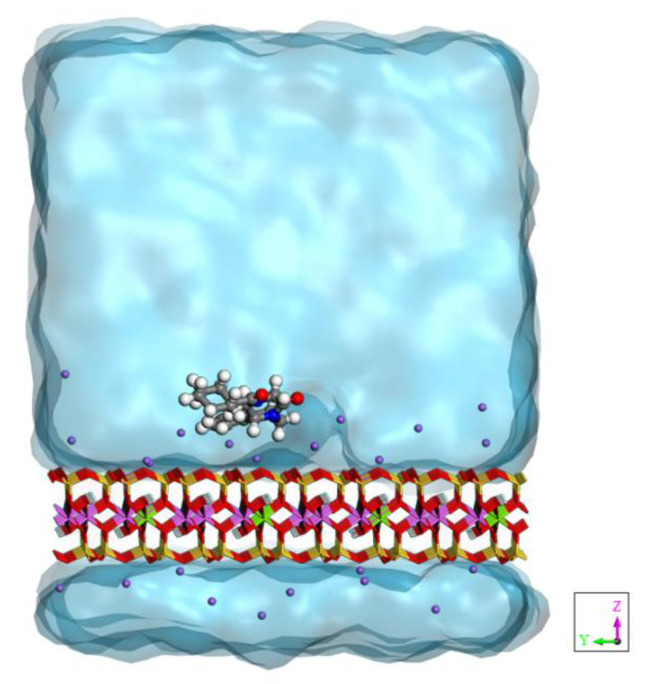
Model of praziquantel adsorbed on the montmorillonite surface in aqueous solution.

The montmorillonite surface was modeled as a slab, containing 6 × 4 layer unit cells to which periodic boundary conditions were applied in the *x*, *y* plane. The resulting periodically repeated unit in the supercell had stoichiometry Na_24_(Al_76_Mg_20_)(Si_188_Al_4_)O_480_(OH)_96_. The drug was positioned on the layer surface, and it was immersed in a bath of 1300 water molecules. The periodicity along the direction *z* perpendicular to the surface was 45.90 Å (Figure 1). 

#### 2.1.2. Model System for the Case of the Interlayer Adsorbed Drug

**Figure 2:**
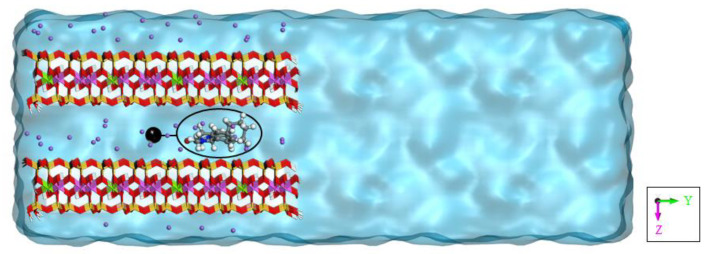
Model of praziquantel adsorbed in the interlayer space of montmorillonite in aqueous solution. Here, only some of the waters in *y* direction are shown. The geometrical descriptor used in the GAMBES and OPES_f_ simulations is highlighted, corresponding to the *y*-component of the vector, connecting the center of mass of the drug molecule and a fixed point in the middle of the clay interlayer space (X^0^) (see text).

We simulated a molecule of praziquantel adsorbed in a space between two montmorillonite layers immersed in water (Figure 2). In particular, a 6 × 4 × 2 supercell of montmorillonite was created with the same composition as a previous experimental work [19]. The edges of both layers were cleaved along the (010) and (0$\bar{1}$0) planes to break the periodicity of the layers along the *y* direction. The valence of the oxygen atoms was completed by adding hydrogen atoms. During the simulations, the structural integrity of the clay edges was preserved. The terminating hydrogens were assigned a charge of +0.338 to neutralize the total charge of the clay, also taking into account the negative structural charge of the system. Therefore, the chemical formula of the resulting montmorillonite crystal is Na_48_(Al_152_Mg_40_)(Si_376_Al_8_)O_900_(OH)_312_·96H_2_O. Both layers are identical, and each interlayer space has 48 waters, that is, 2 waters per sodium according to experiments [19]. In one of the interlayer spaces, the praziquantel molecule was adsorbed and 2881 water molecules were placed outside the clay, as shown in Figure 2. Periodic boundary conditions were applied. The box size was L_x_ = 30.96, L_y_ = 128.06, L_z_ = 30.00, α = 90.00, β = 100.46 and γ = 90.00 (distances in Å and angles in °). 

Setting up the system for the study of the drug release from the interlayer region was difficult, due to the small system size and the limitation of the force field. The main problem was that when we set up the simulations some of the counterions left the interlayer region and swelling started, as the counterions were essential for holding together the layers. To avoid this unwanted effect, we limited the interlayer distances. However, despite this precaution, a relative sliding of the two layers was observed. Since we deemed this to be an effect of the system’s finite size, it is unlikely to take place in macroscopic systems. This also forced us to fix this degree of freedom. An account of the attempt made can be found in the Supplementary Materials.

### 2.2. Computational Details

The simulations were driven by the LAMMPS [38] suite of programs, interfaced with the metadynamics plugin PLUMED [39]. The force field used was the consistent valence force field, also called cvff interface (CVFF) [40,41], that describes the interaction of layered phyllosilicates with organic compounds. The atomic charges of the montmorillonite were set as in Heinz and Suter [42]. This set up has been used elsewhere to describe the clay structure, and that of organic molecules [21,43,44]. All simulations were performed at the physiological temperature of 310 K.

The equilibration of the system with the drug adsorbed on the clay’s surface consisted of 10 ps NPT dynamic simulations, followed by another 10 ps in the NVT. Subsequently, to determine the release time, we collected statistics from 10 unbiased simulations of 1 ns long, using the orthorhombic version of the Parrinello-Rahman barostat [45].

In the case of the intercalated drug, after a complex equilibration to stabilize the system (see Supplementary Materials), we fixed the layers of the clay (zero forces and zero velocities) to prevent their movement. The value used for the basal spacing *d*(001) was 16 Å. It corresponds to the *d*(001) measured experimentally for the praziquantel-montmorillonite systems [19,20]. Subsequently, to calculate the drug release kinetics, we ran 25 independent biased NVT simulations up to 100 ns long.

In GAMBES, we used only one descriptor d that is the *y*-component of the vector, connecting the center of mass of the drug molecule and a fixed point in the middle of the clay interlayer space (X^0^). The biased simulations started with the drug at X^0^, from which it diffuses before escaping. The static bias $V\left(d\right)$ was constructed to act only on this known state and to drive the drug release process. To limit the bias deposition, the energy cutoff related to the ϵ parameter was 7 kcal/mol. This value allowed the drug release.

**Figure 3:**
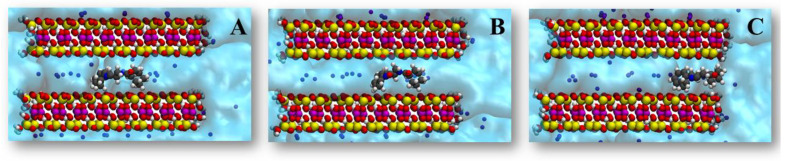
Different initial structures with the praziquantel placed in the center (**A**), between the center and the edge (**B**), and in the edge (**C**), of the montmorillonite interlayer space.

In OPES_f_, we used as CV s the same variable as in GAMBES. We prevented depositing bias in the region *y* > 8.5 or < −8.5 Å (EXCLUDED_REGION). To calculate τ of different structures and then the diffusion coefficient, the cutoff was 7 kcal/mol when the starting point of the drug molecule was at X^0^ (Figure 3A), and also when it was between X^0^ and the edge of the clay (Figure 3B). No bias was required when it was in the edge of the interlayer space (Figure 3C).

To choose these cutoffs, we previously performed several biased simulations, starting from low values and increasing them progressively, until we obtained those that allowed us to observe drug release during the simulation time. In addition, we also controlled the convergence of the bias in the basin of the initial state before the transition occurs. This allows us to obtain accurate kinetics. Lower cutoffs would not release the drug and higher values would exceed the free energy barrier, and might decrease the accuracy of the kinetic estimation, as it would overfill the basin.

In the Supplementary Materials, we show that the GAMBES and OPES_f_ methods give very close results. However, in this application, OPES_f_ appears to be more efficient. The results presented in the main text are based on OPES_f_.

## 3. Results and Discussion

### 3.1. Drug Adsorbed on the Clay’s Surface

We performed ten unbiased simulations of praziquantel adsorbed on the montmorillonite surface. The drug showed a fast desorption from the clay into the water, with a computed release time of 363 ps (see Table 1).

**Figure 4:**
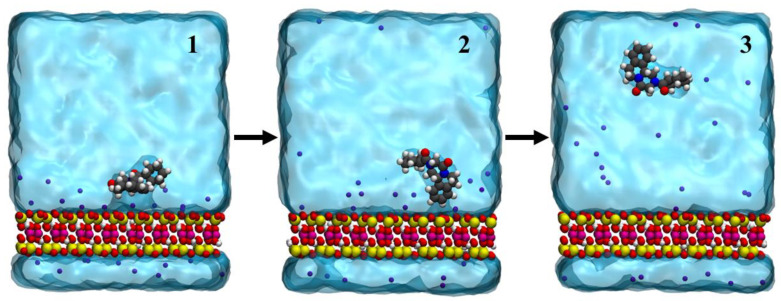
Desorption of praziquantel from montmorillonite surface in aqueous solution, obtained from standard molecular dynamics simulations.

Selected snapshots from a representative release trajectory are displayed in Figure 4. In the initial structure (Figure 4, panel 1), the drug is adsorbed on the surface of the clay in an orientation almost parallel to the clay surface, and a planar conformation. Subsequently, the drug adopts a perpendicular orientation (Figure 4, panel 2) and a bent conformation. Finally, the praziquantel completely loses its interaction with the montmorillonite surface and the drug is desorbed (Figure 4, panel 3). In ~71% of the cases, the aromatic ring is the last to be released. In the other cases, the aliphatic part is released last instead.

These results indicate that in the physiological environment, the praziquantel molecules will be immediately released.

### 3.2. Drug Adsorbed in the Clay’s Interlayer Space

In this case, the drug release process takes place in two steps. In the first, the drug diffuses inside the clay. In the second step, it is released from the edge of the clay to the water. To characterize the kinetics of both steps, the simulations were performed with the drug starting from the three different positions displayed in Figure 3. OPES_f_ was needed when the molecule was inside the clay and therefore we ran two sets of 25 biased simulations from the structures of Figure 3A, B. In the case of the drug positioned at the edge (Figure 3C), observing the release did not require enhanced sampling and we carried out 25 unbiased simulations.

Table 2 shows the time that praziquantel takes to exit to the water solution from the three situations. As can be seen, when it is initially located at the center X^0^ (Figure 3A), τ = 200 µs. This *τ* decreases to 54.4 µs when the molecule starts at a position closer to the edge (Figure 3B). Finally, at the edge, we obtained a *τ* value of only 5.47 ns (Figure 3C).

With these results, we observed that diffusion within the clay is the slowest process. To get an estimate of the diffusion coefficient *D* inside the clay, we perform two different calculations that start from two different distances from the edge *y1* and *y2*, where *y2* (Figure 3A) is further away from the rim than *y1* (Figure 3B). If the exit time in these two cases is *t1* and *t2*, then we use $\;D\;\sim \;\frac{{\left(y^{2}-y^{1}\right)}^{2}}{t^{2}-t^{1}}$. While not rigorous, this provides a rough estimate of this important parameter. The calculated D was 1.10 × 10^−11^ cm^2^ s^−1^. This value is consistent with previous experimental results on a similar organic molecule (tryptophan) trapped in a clay-based material (D ~ 5 × 10^−11^ cm^2^ s^−1^) [46]. It is five orders of magnitude smaller than the diffusion coefficient of praziquantel dissolved in water [47,48]. 

The computed kinetics and diffusion rate can be roughly compared with our experimental data on the praziquantel–montmorillonite release profiles, measured in sink conditions [20]. At the first data measurement at 10 min, >80% of praziquantel was already released in the aqueous solution (pH 6.8). This means that this time must be considered as a high upper limit. On the other hand, the clay dimensions can reach the μm scale [49,50,51]. Even though estimating the diffusion rate occurring in those drug release experimental conditions is hard, due to the complexity of the process (several layer lengths, drug-clay adsorption at distinct regions, etc.), a value in the order of 10^−11^ cm^2^ s^−1^ is compatible with such a scenario. The use of a methodology in the present work, that has been demonstrated in protein–ligand studies [24] to be highly accurate, allows us to quantify the diffusion coefficient of the praziquantel release from the interlayer space of montmorillonite as 1.10 × 10^−11^ cm^2^ s^−1^. It agrees with the fast drug release profiles observed in the experiments and complements the experimental perspective.

**Figure 5:**
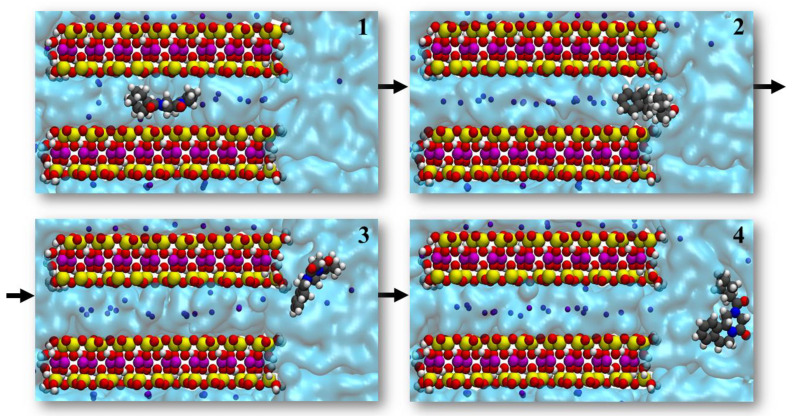
Praziquantel release mechanism from the interlayer space of the montmorillonite in aqueous solution.

Next, we describe the praziquantel release mechanism. The drug is initially in a parallel orientation, interacting with both layers of the clay. Then, it diffuses to the edge, always keeping this double binding (Figure 5, panels 1 and 2). Throughout the diffusion process, the oxygen of the carbonyl groups interacts with the silicon atoms of the clay surface. In addition, the carbonyl group negative charges are screened by sodium cations and water. However, before exiting, the drug interacts with only one layer (Figure 5, panel 3). Water molecules solvate it, favoring its final release (Figure 5, panel 4). Most of the time (~60% of the cases) the aromatic ring is the last part to be released into the solution. This value is slightly lower than that occurring in the surface model (~71% of the cases). Therefore, it seems that the hydration of the interlayer space favors the aromatic ring to be the last part to leave the interaction with the clay. Once the praziquantel is released, the cations cease to help screen the carbonyl charges, a task that is from now on left to the water molecules.

## 4. Conclusions

This paper shows that despite the shortcoming of the potentials, valuable information can be obtained from molecular dynamics calculations in the drug delivery field, as in the protein–ligand research, where the same methodology has been proven to be accurate [24]. Our main finding is that in the case in which the praziquantel molecule is inserted in the interlayer regions, the rate-limiting step is the drug diffusion toward the water solution. Once the drug is at the layer edge the drug release is extremely fast, of the order of a few hundredth picoseconds. Equally fast is the desorption from the external clay surface. The rapid release of the drug obtained with these calculations is in agreement with previous experiments [19,20] and allows for the deciphering of the mechanism, and detailed kinetics aspects.

This suggests several strategies to modulate the release time. For instance, one could search for ways of controlling the penetration length inside the clay. Attempts could also be made at regulating the interlayer distance, by means of appropriate spacers [52,53,54,55,56], or by using other clays with different interlayer spacing [13,57,58,59,60]. In the latter two cases, our approach will allow, in future studies, different candidates to be screened before performing the experiments. As shown in this work, this methodology requires in the first step to prepare and equilibrate, by means of molecular dynamics, a system that simulates the drug adsorbed in the excipient in aqueous solution. Next, enhanced sampling dynamics are used to push the drug from inside the excipient, to the aqueous solution, and accelerate this process which otherwise would not be possible to model by conventional molecular dynamics. From the outcomes of these computations, the diffusion and release times can be obtained.

## Figures and Tables

**Table 1:** Drug release time (τ ) and rate (*k* = 1/τ ). *p*-value measures the quality of the fit using the Kolmogorov–Smirnov analysis. We also present the average release time *µ* and its standard deviation *σ*.

	*τ* (10^−12^ s)	*k* (10^9^ s^−1^)	*p*-Value	*µ ± σ* (10^−12^ s)
Surface, MD	363	2.76	0.76	344 ± 218

**Table 2:** Drug release time (τ ) and rate (*k* = 1/τ ) for structures A, B and C of Figure 3. *p*-value measures the quality of the fit using the Kolmogorov–Smirnov analysis. We also present the average release time *µ* and its standard deviation *σ*.

	$\tau \;(10^{-6}\;s)$	*k* (10^6^ s^−1^)	*p*-Value	*µ ± σ* (10^−6^ s)
A, OPES_f_	200.0	0.005	0.76	198.0 ± 176.0
B, OPES_f_	54.4	0.018	0.41	55.4 ± 64.5
C, MD	0.00547	182.8	0.42	0.00536 ± 0.00393

## Data Availability

Not applicable.

## Supplementary Materials

The following supporting information can be downloaded at: https://www.mdpi.com/article/10.3390/pharmaceutics14122586/s1, Table S1: Praziquantel release time (τ) and rate (*k* = 1/τ). *p*-value measures the quality of the fit using the Kolmogorov–Smirnov analysis. *µ* and *σ* are the average and the standard deviation of the data, respectively; Figure S1: Drug release mechanism from the interlayer space of the montmorillonite with a high swelling in aqueous solution; Table S2: Drug release time (*τ*) and rate (*k* = 1/*τ*) for structures A, B and C of Figure 3. *p*-value measures the quality of the fit using the Kolmogorov–Smirnov analysis. *µ* and *σ* are the average and the standard deviation of the data, respectively; Figure S2: Praziquantel release mechanism from the interlayer space of the montmorillonite with a small swelling in aqueous solution. References [22,23,45,61] have been cited in Supplementary Materials.
