# Supplementary material for: Kinetics of Drug Release from Clay Using Enhanced Sampling Methods
Source: Pharmaceutics. 2022 Nov 24;14(12):2586. doi: 10.3390/pharmaceutics14122586 (PMC9781022; doi:10.3390/pharmaceutics14122586)
Supplement: Supplementary file 1 [file pharmaceutics-14-02586-s001.zip › pharmaceutics-2000555-supplementary.pdf]

pharmaceutics

Supplementary Materials

Kinetics of drug release from clay using enhanced sampling methods

Ana Borrego-Sánchez [1], Jayashrita Debnath [1] and Michele Parrinello [1,*]

TABLE OF CONTENTS

1. Computational details
1.1. *Equilibration of the system with the drug intercalated in the clay*
1.2. *Comparison of the GAMBES and OPESf methods*
1.3. *Clay swelling*
1.4. *Diffusion of sodium cations*

2. Results and Discussion
2.1. Comparison of the GAMBES and OPESf methods
2.2. Clay swelling
2.3. Diffusion of sodium cations

1. Computational details

1.1. Equilibration of the system with the drug intercalated in the clay

Setting up the simulations for the release of the intercalated drug was complicated. The equilibration was achieved in the following manner. The first step was to generate a 50 ps NPT ensemble. This short NPT dynamics allowed the lattice parameters to relax and readjust the cell size to the values L_x=30.97, L_y=145.75 and L_z=24.84 (distances in Å). Then, 250 ps of NVT dynamics simulations were carried out followed by 1 ns NPH simulations using the Parrinello-Rahman barostat [1]. Subsequently, we used an anisotropic version of the Parrinello-Rahman barostat (200 ps) in which the shape of the box is orthorhombic and only the L_y length was allowed to change. Eventually, L_y was set to a value of 131.06 Å. Next, 245 ps of NVT ensemble dynamics were carried out. Finally, a long NVT ensemble of 10 ns was generated. After this equilibration steps, we selected a structure with a basal spacing of 16 Å, with the sodium cations inside the interlayer space and without slide between the clay's layers.

1.2. Comparison GAMBES and OPES$_f$ methods

For the comparison of GAMBES [2] and OPES$_f$ methods [3,4], the kinetics of the drug release were calculated with the equilibrated system keeping the clay layers free (unlike the system described in the manuscript in which the layers are fixed). We ran 25 independent biased NVT simulations up to 50 ns long with both methods.

Following the OPES$_f$ methodology detailed in the manuscript, in this system the cutoff was 5 kcal/mol when the starting point of the molecule was at the middle of the clay interlayer space (X^0) (Figure 3A of the manuscript) and 2 kcal/mol when it was between X^0 and the edge of the clay (Figure 3B). No bias was required when it was in the edge of the interlayer space (Figure 3C).

1.3. Clay swelling

To investigate the swelling of the clay mineral, the NPH step of the equilibration procedure described in section 1.1 was modified. Specifically, the NPH dynamics using the Parrinello-Rahman barostat were carried out testing 8 distinct times in the range from 0.1 to 2 ns. In this step, the clay swells and the swelling affects the drug release. Therefore, we used different times to obtain structures with different interlayer spacing. After finishing all the steps of the equilibration, we chose two structures, with small and large basal spacing, to test the drug release kinetics in these conditions.

1.4. Diffusion of the sodium cations

To study if the diffusion of the sodium cations affects the swelling of the clay observed during the NPH dynamics (see section 1.3), we performed test calculations maintaining the sodium cations within the interlayer space of the clay. For that, the velocities and forces in the Y axis were set to zero, allowing mobility in the X and Z axes. NPH MD simulations were carried out here with times up to 10 ns.

In addition, this strategy based on maintaining the sodium counterions inside the clay was used to investigate if their diffusion out of the clay affects the drug desorption. In this case, the praziquantel release time was calculated with the sodium cations forced to be inside the clay and following the $OPES_f$ methodology described in the manuscript.

2. Results and Discussion

2.1. Comparison of the GAMBES and $OPES_f$ methods

We performed 25 NVT biased simulations of the model system with a *d*(001) ~ 16 Å, free clay's layers and free sodium cations, and the molecule at X^0. Table S1 shows the kinetics data obtained in these GAMBES and $OPES_f$ simulations. As can be seen, the two computed release times are satisfactorily close to each other and either GAMBES or $OPES_f$ could be used for the overall study of the drug release phenomenon. However, we chose $OPES_f$ that proved to be computationally more efficient in our system.

Table S1. Praziquantel release time (τ) and rate (k = 1/ τ). p-value measures the quality of the fit using the Kolmogorov-Smirnov analysis. μ and σ are the average and the standard deviation of the data, respectively.

	τ (10^{-6} s)	k (10^{6} s^{-1})	p-value	$\mu \pm \sigma$ (10^{-6} s)
**GAMBES**	6.37	0.16	0.93	5.94 ± 4.58
**$OPES_f$**	6.07	0.17	0.72	5.82 ± 3.59

2.2. Clay swelling

The swelling of the montmorillonite and its effect on the drug release process were tested here. For this purpose, we studied the kinetics for the drug release similarly as in the manuscript but maintaining free the clay layers. During the equilibration of section 1.1 with several times of the NPH dynamics, different degrees of swelling were observed, and we shall focus on two situations of interest. The first one (case 1) corresponds to a large basal spacing (large swelling of the clay) in which the drug molecule is released from the clay without the need of biasing the system. The second type of system (case 2) has a small basal spacing (small swelling). In this case, the drug does not go out with standard MD.

In case 1, the system has a *d*(001) basal spacing ≥ 18 Å. This system is similar to the surface model, but it is affected here by steric interactions with the second layer. The praziquantel molecule is released in less than 10 nanoseconds. The release mechanism is illustrated in Figure S1. In this case, normally at the beginning of the dynamics the drug is placed also parallel to the clay surface in a flat conformation, and the praziquantel interacts only with one layer of the montmorillonite (Figure S1, panel 1). Later, it is placed

vertically with respect to the clay, the aliphatic part interacting with one layer and the aromatic part with the other layer (Figure S1, panel 2). Subsequently, the drug goes to the edge of the clay and rotates, placing the aromatic part (in ~68 % of the cases) towards one layer of the clay and losing the interaction with the other layer (Figure S1, panel 3). The aromatic part interacts with the clay edge. Finally, the drug is released into the aqueous solution (Figure S1, panel 4).

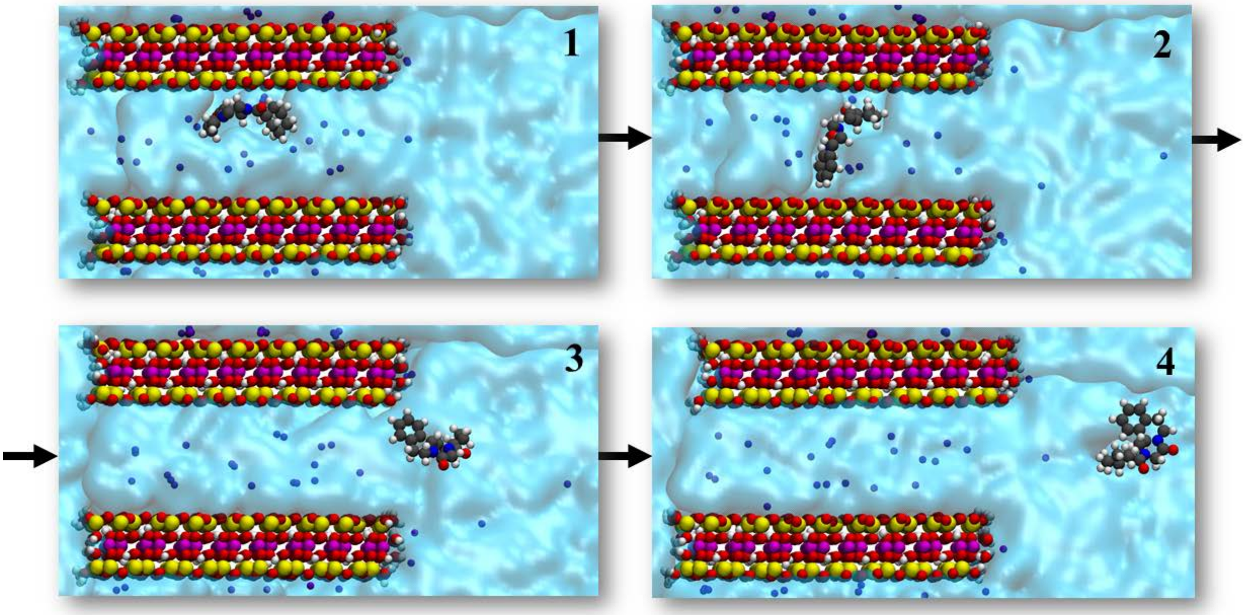

Figure S1. Drug release mechanism from the interlayer space of the montmorillonite with a high swelling in aqueous solution.

Regarding case 2, the system has a *d*(001) basal spacing of 16 Å. In Table S2, we summarize the results obtained using $OPES_f$. As can be seen, when the drug is initially at X^0 (Figure 3A), the τ = 6.07 μs. This τ decreases to 0.86 μs when the drug is initially placed between X^0 and the edge of the layers (Figure 3B). Finally, in the case of the drug located at the edge of the clay, the release did not require the use of enhanced sampling methods and with MD the calculated τ was 2.87 ns (Figure 3C).

Table S2. Drug release time (τ) and rate (k = 1/ τ) for structures A, B and C of Figure 3. p-value measures the quality of the fit using the Kolmogorov-Smirnov analysis. μ and σ are the average and the standard deviation of the data, respectively.

	**τ (10^{-6} s)**	**k (10^{6} s^{-1})**	**p-value**	**$\mu \pm \sigma$ (10^{-6} s)**
**A, $OPES_f$**	6.07	0.17	0.72	5.82 ± 3.59
**B, $OPES_f$**	0.96	1.40	0.46	0.92 ± 0.79
**C, MD**	0.00287	368.0	0.42	0.00274± 0.00226

The diffusion coefficient *D* inside the clay was estimated as we described in the manuscript ($D \sim \frac{(y^2-y^1)^2}{t^2-t^1}$). The calculated *D* here is $3.13 \cdot 10^{-18}$ m^2 s^{-1}. Therefore, when the clay layers are free, the drug release is faster than with the fixed layers. The movement of the layers favors the release of the drug.

In Figure S2, selected snapshots from a representative release trajectory of case 2 are displayed. In the simulations, the drug initially diffuses to the edge (Figure S2, panels 1 and 2). Then, a slide of the layers occurs and the drug begins to be released, maintaining the interaction with the edge of one layer (Figure S2, panel 3). At this point, the drug is

more surrounded by water and its release is favored. Subsequently, praziquantel is completely released (Figure S2, panel 4). Once more, we see that the aromatic ring of the praziquantel is the last part of being released into the water.

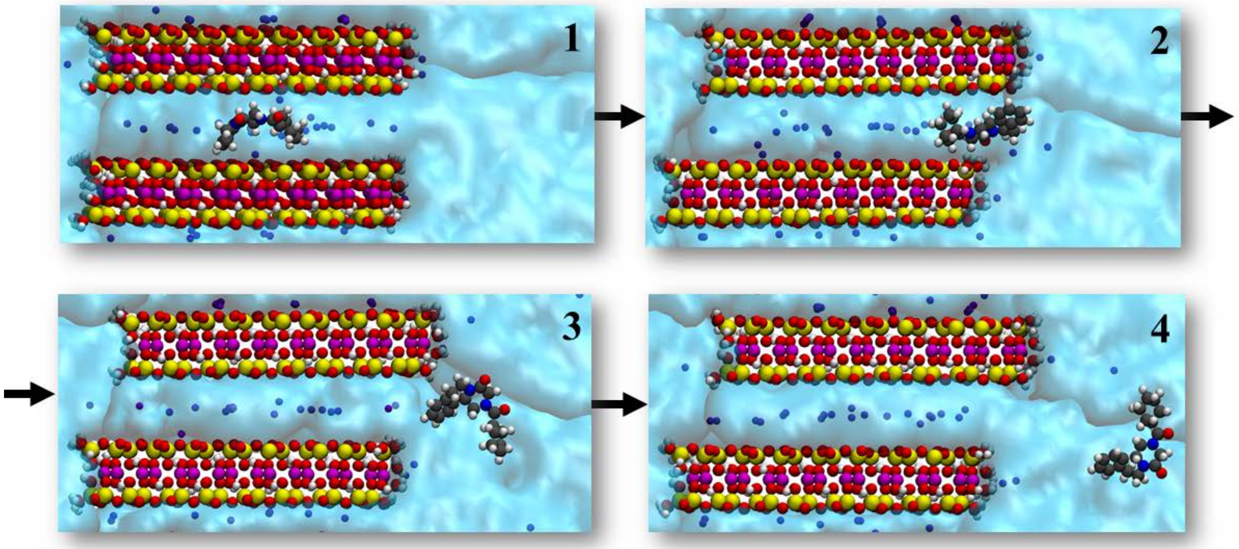

Figure S2. Praziquantel release mechanism from the interlayer space of the montmorillonite with a small swelling in aqueous solution.

Overall, we observe here that the fast drug release is associated to the swelling and slide motions of the layers detected in the conditions of free layers. These motions are unlikely to take place in macroscopic systems. Therefore, these results confirm that the model and kinetics that best approximates the real conditions are those with fixed layers that avoids these unwanted effects. These data are shown in the manuscript.

2.3. Diffusion of sodium cations

Another test was performed to see if the outflow of the sodium cations into the water affects the swelling of the clay. For that, the counterions were not allowed to escape of the interlayer space, and the clay layers were kept free. During the MD simulations, no swelling of the clay was observed. These results corroborate that the diffusion of the sodium cations to water is responsible for the clay swelling described in the previous section.

Finally, we determined the kinetics of the release process with the sodium cations forced to be inside the clay and free layers. Following the $OPES_f$ methodology as in the manuscript, the praziquantel release time obtained here is $\tau = 175$ μs. This result is similar as that obtained in the manuscript with free sodium cations and fixed layers ($\tau = 200$ μs). Therefore, the mobility of the sodium cations does not affect the drug release.

Supplementary References

1. Parrinello, M.; Rahman, A. Polymorphic transitions in single crystals: A new molecular dynamics method. *J. Appl. Phys.* 1981, 52, 7182–7190. https://doi.org/10.1063/1.328693
2. Debnath, J.; Parrinello, M. Gaussian Mixture-Based Enhanced Sampling for Statics and Dynamics. *J. Phys. Chem. Lett.* **2020**, 11, 5076–5080. https://doi.org/10.1021/acs.jpclett.0c01125
3. Invernizzi, M.; Parrinello, M. Rethinking Metadynamics: From Bias Potentials to Probability Distributions. *J. Phys. Chem. Lett.* **2020**, 11, 2731–2736. https://doi.org/10.1021/acs.jpclett.0c00497
4. Ray, D.; Ansari, N.; Rizzi, V.; Invernizzi, M.; Parrinello, M. Rare Event Kinetics from Adaptive Bias Enhanced Sampling. *J. Chem. Theory Comput.* **2022**, *in press*. https://doi.org/10.1021/acs.jctc.2c00806
